# Thyroid function in children with growth hormone (GH) deficiency during the initial phase of GH replacement therapy - clinical implications

**DOI:** 10.1186/1756-6614-3-2

**Published:** 2010-03-22

**Authors:** Joanna Smyczynska, Maciej Hilczer, Renata Stawerska, Andrzej Lewinski

**Affiliations:** 1 Department of Paediatric Endocrinology, Medical University, Lodz, Poland; 2 Department of Endocrinology and Metabolic Diseases, Polish Mother's Memorial Hospital - Research Institute, Lodz, Poland; 3 Department of Endocrinology and Metabolic Diseases, Medical University, Lodz, Poland

## Abstract

**Background:**

Normal thyroid hormone secretion or appropriate L-thyroxine (L-T_4_) substitution is necessary for the optimal effect of the growth hormone (GH) administration on growth rate. The decrease of free thyroxine (FT_4_) levels at recombinant human GH (rhGH) therapy onset has been reported in several studies. The aim of the present study was to evaluate the effect of rhGH administration on thyrotropin (TSH) and FT_4 _serum concentrations in children with GH deficiency (GHD) during the 1st year of therapy, as well as to assess potential indications to thyroid hormone supplementation in them.

**Patients and methods:**

The analysis involved data of 75 children (59 boys, 16 girls) with disorders of GH secretion (GHD, neurosecretory dysfunction - NSD) and partial GH inactivity (inactGH), who were treated with rhGH for - at least - one year. In all the children, body height and height velocity (HV) were assessed before and after 1 year of therapy, while TSH, FT_4_, IGF-I and IGFBP-3 before treatment and after 3-6 months and 1 year of treatment. In the patients, who revealed hypothyroidism (HypoT), an appropriate L-T_4 _substitution was introduced immediately. The incidence of HypoT, occurring during the initial phase of rhGH therapy, was assessed, as well as its influence on the therapy effectiveness.

**Results:**

Before rhGH substitution, there were no significant differences in either auxological indices or TSH and FT_4 _secretion, or IGF-I concentration and its bioavailability among the groups of patients. During the initial 3-6 months of rhGH administration, a significant decrease of FT_4 _serum concentration, together with a significant increase of IGF-I SDS and IGF-I/IGFBP-3 molar ratio was observed in all the studied groups. In 17 children, HypoT was diagnosed and L-T_4 _substitution was administered. Despite similar IGF-I secretion increase, the improvement of HV presented significantly lower in children with HypoT than in those who remained euthyroid all the time.

**Conclusions:**

The incidence of HypoT during the initial phase of GH treatment in children with GHD and the negative effect of even transient thyroid hormone deficiency on the growth rate should be taken into account.

## Background

Growth hormone (GH) deficiency (GHD) in children with short stature is an unchallenged indication to the therapy with recombinant human GH (rhGH). The main goal of the treatment is to increase patients' height velocity (HV) and to improve the attained final height (FH). The most important peripheral mediator of GH activity is the insulin-like growth factor-I (IGF-I). The insulin-like growth factor binding protein-3 (IGFBP-3) is the main carrier protein binding to IGF-I in plasma, thus determining its bioavailability. Besides, either normal thyroid hormone secretion or appropriate substitution of L-thyroxine (L-T_4_) is necessary for the optimal effect of both endogenous GH and rhGH substitution on the growth rate.

The relationships between GH secretion and thyroid function, as well as the effects of rhGH administration on thyroid hormone levels have been the subject of numerous studies. The data of Cacciari et al. [1], presented 30 years ago, indicated that the risk of inducing an alteration in thyroid function in hypopituitary patients during rhGH therapy was only slight and that the abnormal values of thyroxine (T_4_) and triiodothyronine (T_3_) returned to normal limits during follow-up. Next, Gács and Bános [2] reported that rhGH therapy in children with idiopathic GHD reduced T_4 _secretion and affected the peripheral metabolism of thyroid hormones, resulting in an increase of T_3_. In 1994, Jørgensen et al. [3] reported that, in GH-deficient adults, rhGH administration stimulated peripheral T_4 _to T_3 _conversion in a dose-dependent manner and influenced circadian rhythm of thyrotropin (TSH) secretion. Moreover, in some of those patients before rhGH administration, serum T_3 _levels were subnormal despite T_4 _substitution and normalised during the therapy. As it was shown that rhGH administration might induce a fall in serum T_4_, it seemed probable that GHD could mask secondary hypothyroidism in some patients with hypopituitarism. Recently, Agha et al. [4] proved that rhGH administration really led to „unmasking” hypotyroidism in hypopituitary adults. Similar were the observations of Losa et al. [5], who reported that, in adults with GHD, administration of rhGH therapy was associated with a significant decrease of free T_4 _(FT_4_) in first 6 months of treatment.

First reports, concerning the effects of rhGH therapy on thyroid hormone levels in children also confirmed an increase of extrathyroidal conversion of T_4 _to T_3 _during the therapy [6]. Conversely than described for adults [5], as early as in 1994, Laurberg et al. [7] stated that children with GHD, evaluated thoroughly to exclude secondary thyroid failure before rhGH administration, did not develop hypothyroidism during rhGH substitution.

Up to now, several interesting studies have been published on long-term effects of rhGH replacement therapy on thyroid function, both in adults [8-10] and in children [11-18].

The aim of current study was to evaluate the effect of rhGH substitution on TSH and FT_4 _serum concentrations in children with GHD during the 1^st ^year of therapy, as well as to assess potential indications to thyroid hormone supplementation in them.

## Patients and methods

The retrospective analysis involved the data of 75 children (59 boys, 16 girls) with GHD, who were qualified to rhGH therapy. At therapy onset, the patients' height was below the 3rd centile, according to Polish reference charts [19], HV was slow (below -1.0 SD/year), bone age was delayed, according to Greulich-Pyle's standards [20]. Thyroid function was normal in most of children (67 cases). In the remaining 8 patients, L-T_4 _supplementation had been administered, due to either hyperthyrotropinemia or relatively low (normal but close to the lower limit of reference range) FT_4 _concentration and pharmacological euthyrodism was then confirmed. In all the children IGF-I and IGFBP-3 secretion was measured in a single blood sample during in morning hours. In most of the children, IGF-I concentration was either decreased or close to lower limit of normal range. It should be mentioned that - though IGF-I is the main peripheral mediator of GH action - children with normal IGF-I secretion may be diagnosed as GH-deficient and qualified to rhGH therapy in the light of current national recommendations, enclosed in the programme of therapy of GHD in children with rhGH [21]. In all the patients, nocturnal GH secretion was assessed during 3 hours after falling asleep (5 samples every 30 minutes from the 60th to the 180th minute) and 2 stimulating tests were performed (with clonidine 0.15 mg/m^2 ^orally and with glucagon 30 μg/kg, not exceeding 1 mg, *i.m*.). The diagnosis of GHD was established when GH peak during nocturnal assessment and in both stimulating tests was below 10 ng/ml. Neurosecretory dysfunction (NSD) was diagnosed in children with normal results of stimulating tests but decreased nocturnal GH secretion (that observation had to be confirmed by documenting decreased GH secretion in prolonged, 6-hour nocturnal profile). In children with decreased IGF-I secretion and normal GH peak (both in nocturnal profile and after pharmacological stimulation), IGF-I generation test was performed after exclusion of other causes of IGF-I deficiency, not related to GH secretion disorders and GH action (like malabsorption syndromes, liver diseases, malnutrition, other severe chronic diseases). Interestingly enough, a good response to rhGH administration - at least, twofold increase of IGF-I secretion, leading to normalisation of its level in plasma - was observed in each case, thus allowing exclusion of GH insensitivity and pointing at decreased bioactivity of endogenous GH in these children. The obtained results of that test supported the indications to rhGH administration in them. Children with either other hormonal deficiencies or severe and/or chronic, growth influencing diseases, as well as those with acquired GHD, were not included to the studied group. All the girls had normal female karyotype.

At rhGH therapy onset, patients' age was 12.2 ± 2.4 years (mean ± SD). The therapy with rhGH in a dose of 0.21 ± 0.02 mg (0.63 ± 0.05 IU)/kg/week was administered for, at least, 1 year. In every patient, TSH and FT_4 _concentration was assessed three times: before the first rhGH injection, after 3-6 months and at after 1 year of treatment; all the blood samples were taken in morning hours. Additionally, IGF-I and IGFP-3 concentrations were measured at the same time points and IGF-I standard deviation score (SDS) for age and sex, as well as IGF-I/IGFBP-3 molar ratio was calculated.

Plasma TSH and FT_4 _concentrations were measured by the electroimmunochemiluminescent method (ECLIA), Roche, Elecsys^®^Systems 1010/2010/modular analytics E170. For TSH, analytical sensitivity was 0.005 μIU/ml, range - up to 100 μIU/ml, intra-assay coefficient of variance (CV) - 1.5-8.6%, accuracy - 1.1-3.0%. Analytical range for FT_4 _was 0.023-7.77 ng/dl, intra-assay CV - 1.4-2.9%, accuracy - 2.7-6.6%.

Growth hormone concentrations were measured by hGH Immulite, DPC assay, calibrated to WHO IRP 80/505 standard, with analytical sensitivity up to 0.01 ng/ml, calibration range up to 40 ng/ml, sensitivity of 0.01 ng/ml, intra-assay CV - 5.3-6.5% and inter-assay CV - 5.5-6.2%.

Both IGF-I and IGFBP-3 concentration was assessed by Immulite, DPC assays. For IGF-I, WHO NIBSC 1^st ^IRR 87/518 standard was applied, with analytical sensitivity 20 ng/ml, calibration range up to 1600 ng/ml, intra-assay CV - 3.1-4.3% and inter-assay CV - 5.8-8.4%. For comparison among children of different age and sex, IGF-I concentrations were expressed as IGF-I SDS, according to DPC reference data. The assay for IGFBP-3 assessment was calibrated to WHO NIBSC Reagent 93/560 standard, with analytical sensitivity 0.02 μg/ml, the calibration range up to 426 μg/ml, the intra-assay CV - 3.5-5.6% and the total CV - 7.5-9.9%. For calculation of IGF-I/IGFBP-3 molar ratio, the following molecular masses were used: 7.5 kDa for IGF-I and 42.0 kDa for IGFBP-3 [22]. For comparison among children with different age and sex, IGF-I concentrations were expressed as SD score (IGF-I SDS), according to DPC reference data.

Statistical analysis included comparison of thyroid function (FT_4 _and TSH concentrations), IGF-I secretion (as IGF-I SDS) and its bioavailability (expressed as IGF-I/IGFBP-3 molar ratio) in particular time points, before and during rhGH therapy. Non-parametric Wilcoxon's test for dependent samples was applied, as the distribution of the analysed parameters (assessed with Kolmogorov-Smirnov's test) presented not consistent with normal distribution. The differences among particular subgroups of patients in the same time point were assessed with non-parametric Kruskall-Wallis' test for independent samples. The level of statistical significance was at p < 0.05.

## Results

**Figure 1:**
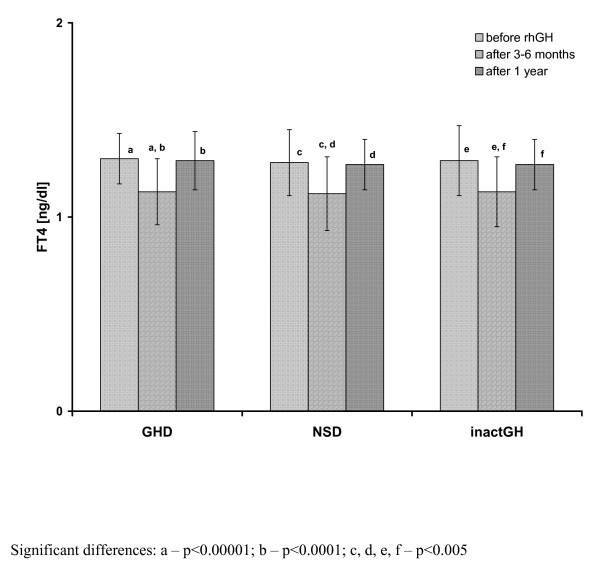
**Free thyroxine serum concentrations before and during rhGH therapy in particular subgroups of patients with respect to the initial diagnosis**.

**Figure 2:**
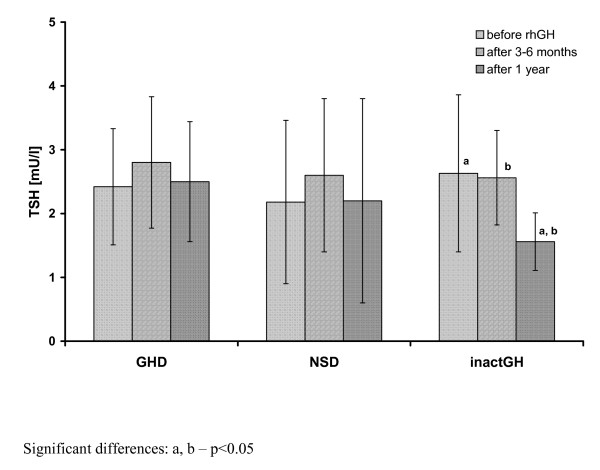
**Thyrotropin secretion before and during rhGH therapy in particular subgroups of patients with respect to the initial diagnosis**.

Before rhGH substitution, there were no significant differences in either auxological indices or TSH and FT_4 _secretion, or IGF-I concentration and its bioavailability among the groups of patients with GHD, NSD and inactGH. Moreover, all the differences among the groups at particular time points (*i.e*. both after 3-6 months and after 1 year of rhGH therapy) still remained insignificant, except for significantly lower TSH in inactGH than in both GHD and NSD after 1 year of rhGH treatment (see Table 1). Interestingly enough, the changes in FT_4 _and TSH concentration were similar in children with previously normal thyroid function (67 cases) and in those on L-T_4 _substitution at rhGH therapy onset (8 cases). Detailed comparisons of the above-mentioned groups of children are presented in Table 2. Among the patients who had L-T_4 _substitution, administered before rhGH therapy onset, the decrease of FT_4 _below the lower limit of normal range was observed in 2 out of 8 cases and in those children, an increased dose of L-T_4 _was necessary to restore euthyroidism. A significant decrease of FT_4 _serum concentration was observed during the initial 3-6 months of rhGH administration, together with insignificant increase of TSH in all the studied groups, as well as in particular subgroups of patients. For more detailed data see Table 1 and Figure 1 and Figure 2.

**Table 1:** Selected auxological and hormonal data of the patients with respect to the initial diagnosis

	Diagnosis			p
		
	GHD	NSD	inactGH	
	
n	36	23	16	
**Before rhGH administration**				

age [years]	12.0 ± 2.4	12.0 ± 2.9	12.9 ± 1.4	0.56

height SDS	-2.59 ± 0.53	-2.91 ± 0.68	-2.68 ± 0.49	0.57

TSH [mU/l]	2.42 ± 0.91	2.18 ± 1.02	2,63 ± 0.94	0.69

FT_4 _[ng/dl]	1.31 ± 0.13	1.28 ± 0.17	1.29 ± 0.15	0.45

IGF-I SDS	-1.77 ± 0.92	-2.18 ± 1.23	-2.36 ± 1.06	0.58

IGF-I/IGFBP-3	0.21 ± 0.08	0.16 ± 0.05	0.16 ± 0.05	0.31

**After 3-6 months of rhGH therapy**				

TSH [mU/l]	2.80 ± 1.28	2.60 ± 1.20	2.56 ± 1.60	0.77

FT_4 _[ng/dl]	1.13 ± 0.17	1.13 ± 0.19	1.13 ± 0.13	0.73

IGF-I SDS	0.33 ± 0.99	0.40 ± 0.89	0.33 ± 0.74	0.38

IGF-I/IGFBP-3	0.38 ± 0.13	0.37 ± 0.11	0.38 ± 0.11	0.86

**After 1 year of rhGH therapy**				

TSH [mU/l]	2.51 ± 1.23	2.15 ± 0.74	1.56 ± 0.45	**0.005**

FT_4 _[ng/dl]	1.29 ± 0.18	1.27 ± 0.18	1.27 ± 0.13	0.72

IGF-I SDS	0.78 ± 0.88	0.62 ± 0.69	0.45 ± 0.83	0.72

IGF-I/IGFBP-3	0.43 ± 0.12	0.41 ± 0.11	0.39 ± 0.09	0.86

**Table 2:** Selected auxological and hormonal data in euthyroid vs. previously L-T_4 _treated patients

	euthyroid	L-T_4 _treated	p
	
n	67	8	
**Before rhGH administration**			

age [years]	12.3 ± 2.2	10.8 ± 3.6	0.15

height SDS	-2.65 ± 0.54	-3.13 ± 0,76	0.07

HV [cm/year]	3.6 ± 0.9	3.8 ± 1.0	0.71

TSH [mU/l]	2.42 ± 0.91	2.18 ± 1.02	0.27

FT_4 _[ng/dl]	1.26 ± 0.15	1.30 ± 0.15	0.97

IGF-I SDS	-2.00 ± 0.98	-2.23 ± 1.72	0.87

IGF-I/IGFBP-3	0.18 ± 0.06	0.19 ± 0.11	0.97

**After 3-6 months of rhGH therapy**			

TSH [mU/l]	2.68 ± 1.38	2.75 ± 0.74	0.52

FT_4 _[ng/dl]	1.12 ± 0.13	1.17 ± 0.37	0.16

IGF-I SDS	0.49 ± 0.83	0.16 ± 1,30	0.29

IGF-I/IGFBP-3	0.39 ± 0.11	0.31 ± 0.13	0.21

**After 1 year of rhGH therapy**			

HV [cm/year]	9.8 ± 2.1	11.7 ± 1.1	0.01

TSH [mU/l]	2.14 ± 0.90	2.94 ± 1.98	0.27

FT_4 _[ng/dl]	1.29 ± 0.16	1.21 ± 0.22	0.55

IGF-I SDS	0.67 ± 0.77	0.61 ± 1.31	0.72

IGF-I/IGFBP-3	0.42 ± 0.11	0.37 ± 0.15	0.64

**Figure 3:**
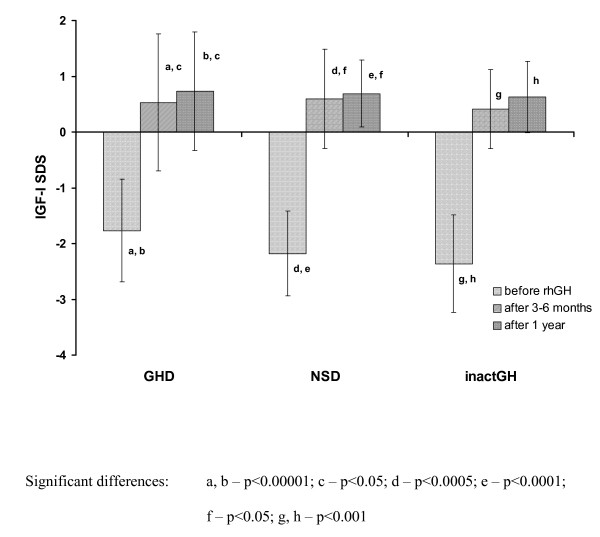
**IGF-I secretion (expressed as IGF-I SDS for age and sex) before and during rhGH therapy in particular subgroups of patients with respect to the initial diagnosis**.

**Figure 4:**
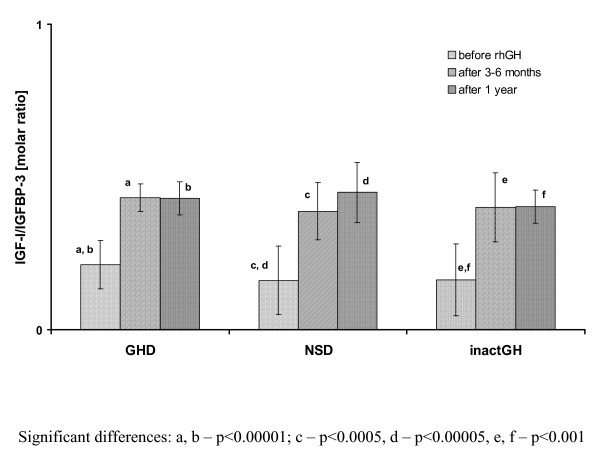
**IGF-I bioavailability (expressed as IGF-I/IGFBP-3 molar ratio) before and during rhGH therapy in particular subgroups of patients with respect to the initial diagnosis**.

Simultaneously, a significant increase of IGF-I SDS was observed not only on the 3-6th month of rhGH therapy, with respect to pre-treatment values, but also after 1 year of therapy vs. the values obtained on the 3-6th month of treatment. Significant differences in IGF-I SDS in particular time points were observed for all the subgroups, expect for that between the values on the 3-6th month and after 1 year in the subgroup with inactGH. Similarly, a significant increase of IGF-I/IGFBP-3 molar ratio was observed on the 3-6th month of rhGH therapy, with respect to pre-treatment values, however, with only a very slight further increase (insignificant) after 1 year of therapy, both in all the studied groups and in the subgroups of patients. The more detailed data are presented in Figure 3 and Figure 4. Interestingly enough, there were no significant differences in either IGF-I SDS or IGF-I/IGFBP-3 molar ratio, observed among the particular subgroups of children at any time point (see Table 1).

According to our observations, the therapy with rhGH led to a similar increase of IGF-I secretion and of its bioavailability in the patients with different forms of disorders of GH secretion and activity. During the initial phase of rhGH replacement therapy, a transient decrease of FT_4 _concentration was observed in all the subgroups of patients, in most subjects being connected with an increase of TSH secretion. Spontaneous normalisation of FT_4 _concentration on the level close to the values obtained before rhGH administration, observed in the majority of patients, presented parallel to further increase of IGF-I secretion.

In 17 children, out of 67 previously untreated with L-T_4 _(25.4%), either FT_4 _concentration decreased below the lower limit of normal range (8 cases) or TSH increased above the upper limit of the normal range (8 cases), or both (1 case). In those patients, L-T_4 _therapy was administered just after the diagnosis of hypothyroidism was established, in the initial daily dose of 25 μg, individually adjusted under control of TSH and FT_4 _levels. In the remaining 50 patients, TSH and FT_4 _concentrations returned to pretreatment values at the end of the 1st year of rhGH administration. Thus, in all the children both TSH and FT_4 _concentrations after 1 year of rhGH therapy were normal. It should be reminded that all the children were euthyroid at rhGH therapy onset.

**Figure 5:**
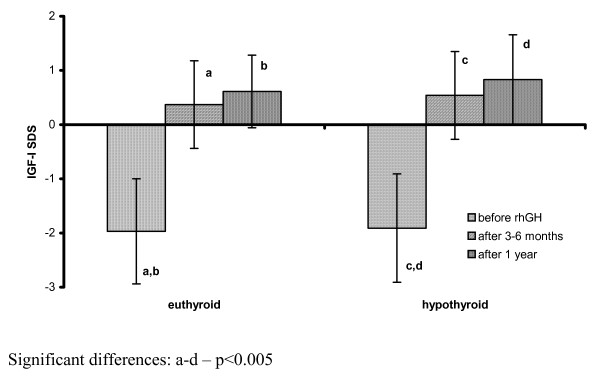
**IGF-I secretion (expressed as IGF-I SDS for age and sex) before and during rhGH therapy in the patients divided according to the thyroid function during the initial phase of rhGH administration**.

**Figure 6:**
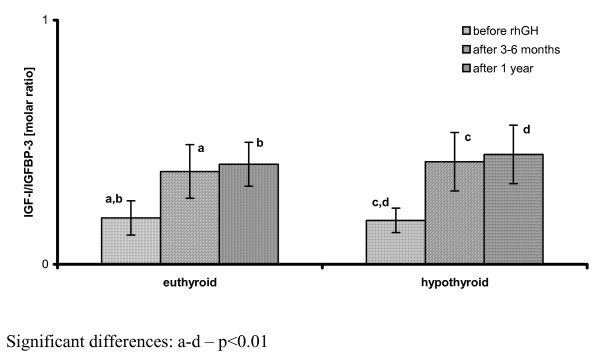
**IGF-I bioavailability (expressed as IGF-I/IGFBP-3 molar ratio) before and during rhGH therapy in the patients divided according to the thyroid function during the initial phase of rhGH administration**.

**Figure 7:**
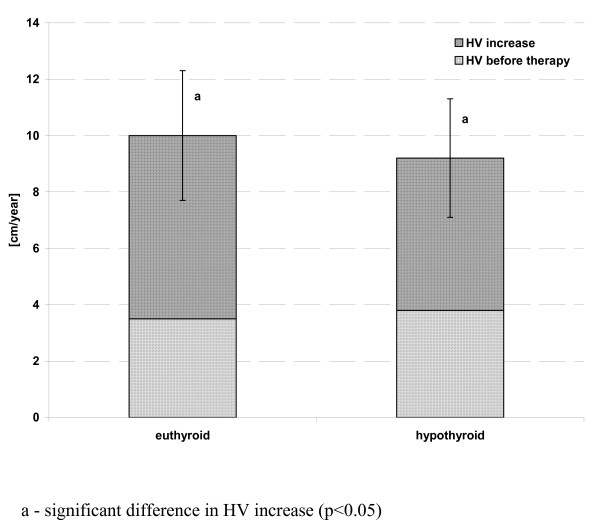
**Height velocity increase in the 1^st ^year of rhGH therapy in the patients divided according to the thyroid function during the initial phase of treatment**.

In the patients, in whom hormonal evaluation after 3-6 months of rhGH administration led to the diagnosis of HypoT (at least in subclinical form), TSH levels were significantly higher, while FT_4 _concentrations - significantly lower than in those children who remained euthyroid during rhGH administration. There were no significant differences between them in either IGF-I SDS or IGF-I/IGFBP-3 molar ratio at that time point. Moreover, after 1 year of rhGH administration there were no significant differences either in IGF-I secretion or in IGF-I/IGFBP-3 molar ratio between the groups of children who were euthyroid all the time and the other one, grouping children who required L-T_4 _substitution. However, it should be emphasized that TSH and FT_4 _levels in all of them were normal at the latter time point. Nevertheless, some differences between the groups were observed and should be mentioned. Surprisingly, IGF-I SDS presented lower (however only insignificantly) in those children who remained euthyroid during all the study period, than in those, who presented with hypothyroidism after 3-6 months of rhGH replacement. Another important observation was a further increase of IGF-I SDS in both groups after 1 year of therapy with respect to the values obtained after 3-6 months. Nevertheless, after 1 year of rhGH therapy, HV improvement (expressed both as the difference and as the ratio between HV during and before the therapy) was significantly lower in those children who were hypothyroid even for a relatively short period of time during the initial phase of rhGH therapy. It should be stressed that L-T_4 _substitution was administered as soon as possible when hypothyroidism was diagnosed. For detailed data see Table 3 and Figure 5, Figure 6 and Figure 7.

**Table 3:** Selected auxological and hormonal data of the patients according to thyroid function during rhGH administration

	euthyroid	hypothyroid	p
	
n	50	17	
**Before rhGH administration**			

age [years]	12.4 ± 2.3	12.3 ± 2.1	0.94

height SDS	-2.60 ± 0.50	-2.77 ± 0.63	0.38

HV [cm/year]	3.5 ± 1.0	3.8 ± 0.9	0.14

TSH [mU/l]	2.22 ± 0.95	2.65 ± 0.92	**0.05**

FT_4 _[ng/dl]	1.33 ± 0.15	1.21 ± 0.11	**0.01**

IGF-I SDS	-2,01 ± 0.97	-1.99 ± 1.00	0.45

IGF-I/IGFBP-3	0.19 ± 0.07	0.18 ± 0.05	0.71

**After 3-6 months of rhGH therapy**			

TSH [mU/l]	2.25 ± 0.92	3.71 ± 1.75	** < 0.001**

FT_4 _[ng/dl]	1.16 ± 0.10	1.01 ± 0.14	** < 0.001**

IGF-I SDS	0.37 ± 0.81	0.54 ± 0.81	0.59

IGF-I/IGFBP-3	0.38 ± 0.11	0.42 ± 0.12	0.36

**After 1 year of rhGH therapy**			

TSH [mU/l]	2.07 ± 0.98	2.16 ± 0.79	0.54

FT_4 _[ng/dl]	1.31 ± 0.15	1.23 ± 0.14	0.11

IGF-I SDS	0.61 ± 0.67	0.83 ± 0.83	0.35

IGF-I/IGFBP-3	0.41 ± 0.09	0.45 ± 0.12	0.84

HV [cm/year]	10.0 ± 2.1	9.3 ± 2.0	0.23

ΔHV [cm/year]	6.5 ± 2.3	5.4 ± 2.1	**0.01**

ΔHV [%]	213 ± 118	154 ± 85	**0.05**

## Discussion

The phenomenon of T_4 _and FT_4 _concentration decrease after rhGH administration in GH-deficient subjects has been reported in several studies [1,2,5,9,12,14]. Also, the return of thyroid function to baseline during follow-up has been quite well documented [13,15,17,18]. Our results confirm previous observations, concerning these problems.

Moreover, it has been suggested that rhGH therapy might disclose previously unrecognised thyroid insufficiency rather than induce hypothyroidism [4,7,14]. The data, concerning the development of central hypothyroidism in terms of rhGH substitution, seem to be rather scarce and non-consistent. In 2007, Agha et al. [7] stated that GHD masked central hypothyroidism in a significant proportion of hypopituitary adults. Conversely, in 2008, Lose et al. [5] reported only a low incidence of hypothyroidism in GH-deficient adults on rhGH substitution. Similar were the observations of Laurberg et al. [7] and Giavoli et al. [16] in GH-deficient children, beginning rhGH therapy. In our study, in 15% of previously euthyroid children, a decrease of FT_4 _level with no adequate increase of TSH was observed, (it should be stressed that children with evident central hypothyroidism before rhGH administration, being a component of multiple pituitary hormone deficiency, were not included in current study). Thus, our observations confirm the phenomenon of „unmasking” central hypothyroidism after rhGH therapy administration in some of children with previous diagnosis of isolated GHD. Changes in TSH secretion during initial period of rhGH substitution are less evident than fluctuations of FT_4 _concentration. Most researchers reported either a lack of significant changes in TSH secretion [1,4,6,9,14,16,18] or a decrease of TSH level in terms of rhGH administration [8,15,17]. The last phenomenon has been explained by an increase of somatostatin (being a natural TSH inhibitor) in the patients on rhGH therapy [15]. In our study, the mean TSH serum concentration presented a slight increase, followed by a recovery to pre-treatment level. It seems that the differences among the results obtained in different studies might be related to different study protocols and analysed time points.

The most frequently quoted mechanism of changes in thyroid hormone levels is GH-mediated increase of peripheral T_4 _to T_3 _deiodination [7-9,12,14]. Moreover, a potential role of IGF-I in stimulating that process has been suggested by Jørgensen et al. [9]. The relationships between GH, IGF-I and thyroid hormone secretion have been subject of numerous studies. More than 25 years ago, in 1983, Chernausek et al. [23] documented that plasma somatomedin C (*i.e*. IGF-I) concentrations were diminished in hypothyroid patients, however, the pathogenesis of this phenomenon remained unclear. Either diminished GH secretion or direct effects of hypothyroidism upon somatomedin production were considered. In early 1990s, Näntö-Salonen et al. [24] stated that the mechanisms of thyroid hormone action on the insulin-like growth factor system were not GH-mediated. Similar were the observations of Inukai et al. [25], concerning the patients with autoimmune thyroid diseases. In 2003, Iglesias et al. [26] stated that hypothyroidism is associated with significant reductions of IGF-1 and IGFBP-3. Next, Purandare et al. [27] documented that in infants with hypothyroidism both total and free IGF-I levels were lower than those in healthy ones and increased significantly after L-T_4 _therapy, while in older children with acquired hypothyroidism they were not significantly lower than in age- and sex-matched controls. However, during L-T_4 _treatment an increase of total IGF-I but not of free IGF-I was observed. Similarly, Bona et al. [28] documented that in the patients with hypothyroidism - both congenital and caused by thyroiditis - L-T_4 _replacement led to physiological increase of IGF-I and IGFBP-3 secretion. Moreover, Schmid et al. [29] showed that, during L-T_4 _replacement, IGF-I and acid-labile subunit secretion increased in the patients with both primary and central hypothyroidism, while IGFBP-3 - only in those with primary hypothyroidism. In 2008, Akin et al. [30] reported that GH-IGF axis was affected in the patients with subclinical hypothyroidism and that L-T_4 _replacement therapy could prevent abnormalities related to GH-IGF axis in them. Moreover, at the same time, Soliman et al. [31] proved that, in children with neglected congenital hypothyroidism, even after long period of hypothyroidism, L-T_4 _replacement improved the growth rate, leading to a partial recovery of GH-IGF-I axis.

In our study, differences were found between the improvement of the growth rate in the patients with normal thyroid function and in those with even transient hypothyroidism. As a matter of fact, a direct effect of thyroid function on IGF-I secretion was not fully confirmed in our study, as there were no significant differences in an increase of IGF-I secretion between the euthyroid children and those, who presented with hypothyroidism during rhGH administration. However, further increase of IGF-I secretion on the same rhGH dose was observed after 1 year of rhGH therapy, with respect to the values obtained after 3-6 months of treatment. This phenomenon might be explained either by the improvement of thyroid function (*i.e*. recovery to pre-rhGH-treatment values of FT_4 _and TSH) or by the appropriate L-T_4 _substitution.

Obligatory L-T_4 _supplementation from the beginning of rhGH therapy in euthyroid patients has not been recommended [17] due to a little evidence for the development of clinically significant hypothyroidism in most of previously euthyroid patients [13] and spontaneous recovery to pre-treatment thyroid function in most cases [13,15,17,18].

Our findings speak for the important role of maintaining euthyroid status of the patients for the best effectiveness of rhGH therapy, as even short-term, transient hypothyroidism presented to be a cause of lower increase of HV in 1st year of rhGH administration. Thus, the incidence of revealing (or "unmasking") hypothyroidism should be taken into account, while starting rhGH administration, as hypothyroidism may worsen the response to the therapy. It seems that either earlier assessment of TSH and FT_4 _concentration after rhGH therapy onset or L-T_4 _administration from the beginning of rhGH therapy in children with normal but relatively low FT_4 _secretion and/or normal but relatively high TSH levels should be taken into account. Further studies seem necessary to fully assess the influence of thyroid function (and thyroid hormone substitution) on the effectiveness of rhGH therapy in children with disorders of GH secretion. It seems also important to establish - if possible - the threshold values of pre-rhGH-treatment TSH and/or FT_4 _levels predictive for revealing hypothyroidism during rhGH administration.

## Conclusions

The incidence of HypoT during the initial phase of rhGH treatment in children with GHD and the negative effect of even transient thyroid hormone deficiency on growth rate should be taken into account while beginning rhGH administration in them.

## Abbreviations

CV: coefficient of variance; FH: final height; FT_4_: free thyroxine; GH: growth hormone; GHD: growth hormone deficiency; HypoT: hypothyroidism; HV: height velocity; IGF-I: insulin-like growth factor-I; IGFBP-3: insulin-like growth factors binding protein-3; L-T_4_: L-thyroxine (levothyroxine); NSD: neurosecretory dysfunction; inactGH: growth hormone inactivity; RhGH: recombinant human growth hormone; SDS: standard deviation score; T_3_: triiodotyronine; T_4_: thyroxine; TSH: thyrotropin.

## Competing interests

The authors declare that they have no competing interests.

## Authors' contributions

JS participated in acquisition of data, performed the statistical evaluation and drafted the manuscript, MH participated in acquisition of data and in design of the study, RS participated in acquisition of data, AL conceived of the study, participated in its design and revised the text of manuscript. All authors read and approved the manuscript.

## Authors' information

AL - Professor; Head of Chair of Endocrinology and Metabolic Diseases, Medical University of Lodz, Poland, Head of Department of Endocrinology and Metabolic Diseases, Polish Mother's Memorial Hospital - Research Institute. MH - Ass. Professor; Head of Department of Pediatric Endocrinology, University of Lodz, Poland. JS - PhD, MD, endocrinologist. RS - PhD, MD, endocrinologist.
